# Dietary fibre effects and the interplay with exogenous carbohydrases in poultry nutrition

**DOI:** 10.1016/j.aninu.2023.09.007

**Published:** 2023-11-29

**Authors:** Michael R. Bedford, Birger Svihus, Aaron J. Cowieson

**Affiliations:** a AB Vista Feed Ingredients Ltd, Marlborough, United Kingdom; b Norwegian University of Life Sciences, Ås, Norway; c DSM Nutritional Products, Kaiseraugst, Switzerland

**Keywords:** Fibre, Carbohydrase, Nutrition, Microbiome, Gastro-intestinal development, Feed

## Abstract

A comprehensive understanding of the role of dietary fibre in non-ruminant animal production is elusive. Equivocal and conflated definitions of fibre coupled with significant analytical complexity, interact with poorly defined host and microbiome relationships. Dietary fibre is known to influence gut development, feed intake and passage rate, nutrient absorption, microbiome taxonomy and function, gut pH, endogenous nutrient loss, environmental sustainability, animal welfare and more. Whilst significant gaps persist in our understanding of fibre in non-ruminant animal production, there is substantial interest in optimizing the fibre fraction of feed to induce high value phenotypes such as improved welfare, live performance and to reduce the environmental footprint of animal production systems. In order to achieve these aspirational goals, it is important to tackle dietary fibre with the same level of scrutiny as is currently done for other critical nutrient classes such as protein, minerals and vitamins. The chemical, mechanical and nutritional role of fibre must be explored at the level of monomeric sugars, oligosaccharides and polysaccharides of varying molecular weight and decoration, and this must be in parallel to standardisation of analytical tools and definitions for speciation. To further complicate subject, exogenous carbohydrases recognise dietary fibre as a focal substrate and have varying capacity to generate lower molecular weight carbohydrates that interact differentially with the host and the enteric microbiome. This short review article will explore the interactive space between dietary fibre and exogenous carbohydrases and will include their nutritional and health effects with emphasis on functional development of the gut, microbiome modulation and host metabolism.

## 1 Introduction and history of carbohydrases with emphasis on poultry production

The first report of the use of an exogenous enzyme blend being added to the diet of commercial poultry is Hervey (1925) and was followed quickly by Clickner and Follwell (1926). In both these cases the enzyme mixtures were crude and the considerable benefits to animal live performance that were generated probably did not stem only from exogenous enzyme activity. Nonetheless, these authors opened interest in a new category of feed additive and stimulated almost 100 years of continuous research and development that has generated a global biotechnology industry with value in excess of $1bn per annum. Feed enzyme use is estimated to save the global animal protein industry an estimated $6bn in nutritional input costs (an average feed cost reduction from the combined use of phytase, protease and carbohydrase). Ironically, this success was despite a prophetic statement from Holst (1926) who, in a review of this nascent technology, declared that “extension workers should be very cautious in advocating the use of artificial enzymes as such, in poultry feeding.”

The nutritional feed enzyme market today comprises three major segments: carbohydrases, proteases and phytases. It is beyond the scope of the present review to describe the mode of action, opportunities and challenges associated with protease or phytase though these will be mentioned in brief. The carbohydrase category is, of course, not homogenous, but comprises an array of mono-component and multi-component carbohydrases across multiple activity classes. Despite this diversity, most exogenous carbohydrases used in commercial swine and poultry production are based on xylanase (endo-1,4-β-xylanase; EC 3.2.1.8) and/or glucanase (endo-1-3(4)-β-glucanase; EC 3.2.1.6). These two activities are the most widely accepted, most frequently guaranteed by enzyme suppliers from a regulatory perspective, most comprehensively studied, and dominant in around 70% of global carbohydrase products. Other notable activities include amylase (not a non-starch polysaccharide [NSP] degrading carbohydrase so will not be discussed in the present review, but a recent review is available (Cowieson et al., 2019)), various pectinases, β-mannanases and α-galactosidases as well as a host of minor and side-activities with variable effects on carbohydrate sidechains and amorphous regions in lignified cellulose. Except for crude enzyme products with diverse side-activities, most carbohydrases used in commercial swine and poultry production are endo-acting (i.e. randomly cleaves interior linkages within the backbone of the fibre structure) and do not generate appreciable concentrations of monomeric sugars. This is partially by design as monomeric sugars from NSP are not universally beneficial for non-ruminant animals. Xylose, for instance, a 5-carbon sugar, is readily absorbed from the intestine of chickens but is not compatible with the Krebs Cycle and so must be excreted in the urine at net cost to the host (Schutte, 1990). Helpfully, reaction products from commercial carbohydrases are typically small oligosaccharides of varying molecular weight and the resulting benefit to the host is usually derived subsequently from fermentation by the intestinal microbiota.

The success of carbohydrases in commercial pig and poultry production stems from appreciable feed cost saving and this is largely associated with increases in metabolizable, digestible, and net energy. This topic will be covered in more detail later, but typical responses range from 30 to 150 kcal/kg, an increase of between 1% and 5% relative to the control diet (Adeola and Cowieson, 2011). Adding this nutrient release value to the enzyme or increasing the energy density of one or more feed ingredients in the least cost formulation ingredient matrix allows nutritionists to capture value by displacing more expensive energy sources from the ration. In addition to this “direct” effect of carbohydrase, there are a range of secondary benefits to animal performance that are generated from improvements in gut health (partially mediated via favourable changes in fermentation patterns in the microbiome), pollutive nature of the faeces, net energy and nutrient requirements, gut motility, development, and the lumen environment etc. Many of these will be discussed in later sections of this review.

The substrate for exogenous carbohydrases is heterogenous and not well defined (Aftab and Bedford, 2018; Choct, 2015a, Choct, 2015b; Nguyen et al., 2021a). This lack of clarity creates challenges for optimization of existing carbohydrase products, e.g. in dose or product selection or in aligning enzyme choice with growth stage, diet type or animal species. These are not unique to carbohydrases but offer a more complex challenge than is the case for phytase which enjoys a more well-defined substrate–enzyme relationship. Despite the challenges in clarifying the role of fibre per se and the interaction between fibre type, origin, solubility profile, molecular weight and exogenous carbohydrases, considerable progress has been made on many of these topics and several will be addressed later.

The purpose of this short review article is to summarise the current state of the art with regard to dietary fibre effects and the interplay between exogenous carbohydrase and dietary fibre. This review will not be entirely exhaustive, but it will highlight key new developments and gaps in understanding that may be fruitful for future research programs. Optimizing the fibre fraction of non-ruminant production animal diets to elicit more consistent live performance outcomes and improving the magnitude and consistency of the effect of exogenous carbohydrases will bring appreciable benefits to the industry, both fiscally and in sustainable animal production.

## 2 What do we mean by dietary fibre?

Fibre is a term that is used generically to describe a very complex and heterogenous group of structures that can have profound effects on digestion and the digestive tract. Fibre can act as a diluent, an antinutrient, a substrate for beneficial bacteria, an immune modulator and as a modulator of intestinal structure and function. The problem with the term “fibre” for nutritionists is that it is not a single entity and depending upon its structure it can be beneficial, detrimental or inert to the performance of the animal. Thus, simply referring to it as “dietary fibre” conveys very little value in describing its potential effects in the animal and this predicament has, in large part, been due to limitations in analysis which prevents assignation of structure to function. A brief history of the development of techniques used to measure fibre contents of diets and ingredients follows and a view with regards to where such analysis may go is offered.

### 2.1 Assays

The term crude fibre refers to the material left after sequential extraction with acid then alkali, assuming that such a treatment leaves only cellulose and lignin in the residue. This method was first introduced more than 200 years ago and is problematic since significant amounts of lignin can be removed by the alkaline extraction process (Norman and Shrikhande, 1935) and the acid process may extract significant amounts of cellulose and leave quantities of pentosans in the residue (Van Soest and McQueen, 1973). However, despite its shortcomings, it is still used today as attempts to remove it completely have failed because it is a simple assay and nutritionists still place some value in it. From a gut function viewpoint, crude fibre offers little value to the monogastric nutritionist due to its variable efficacy in estimating what is in effect supposed to be the non-fermentable fraction.

Neutral detergent fibre (NDF) has largely replaced crude fibre in ruminant nutrition and was considered to be the entire fibre component of the diet (Van Soest and McQueen, 1973). The NDF method relies on measuring the mass of what remains following dissolution of the material in a neutral detergent solution. In addition to cellulose and lignin, this method will also include the majority of the neutral sugar polysaccharides including arabinoxylans, β-glucans, galactans, galactomannans and mannans for example in the residue (Choct, 2015a, Choct, 2015b). However, since most pectic substances are removed in this assay, its ability to determine the fibre content of ingredients rich in pectins, such as legumes, is limited (Jung, 1997). The NDF procedure also fails to remove heat denatured proteins which will inflate the fibre estimate if significant amounts are present.

The acid detergent fibre (ADF) method using an acid detergent is again a gravimetric method that digests all components of the ingredient but leaves the cellulose, lignin and small but variable amounts of xylans and other components. It is this variability which reduces its ability to provide a consistent analysis in different feedstuffs (Jung, 1997). As a result, ADF values are less valuable for cereals and NDF of far lesser value for oilseed meals if the goal is to understand their fibre content. More recent and comprehensive analysis determines total dietary fibre by one of two methods. The first is an enzymatic digestion of non-cell wall carbohydrates and protein and gravimetric determination of the remainder (the AOAC method) followed by subtraction of ash content. The second is also known as the Uppsala method which relies again upon digestion of non-cell wall material followed by acid hydrolysis of the residue and determination of the sugar composition and identity of the resultant monosaccharides (Theander et al., 2020). The AOAC method simply measures the total fibre (which covers all NSP plus lignin) content as a single value whereas the Uppsala method (Theander et al., 2020) will deliver information on the sugar composition of the NSP fraction and in this regard yields far more information than the other methods. Lignin has to be determined separately for an analysis of total fibre content of a feed when using the Uppsala method using the Klason method.

The issue with all of the above methods is that identity of the fibre is at best inferred from its sugar composition even when using the Uppsala method. Greater resolution and value can be achieved by separating the fibre into soluble and insoluble structures, with soluble materials generally being recognised as being more fermentable, but even more sophisticated methods are required to gain functionally relevant information on fibre.

### 2.2 Molecular weight

Sugar composition, backbone structure, identification of substitutions and identification of a fibre as being soluble or not still does not fully explain its functionality. For example, high molecular weight soluble fibres can increase intestinal viscosity and thereby impede digestion (Bedford and Classen, 1992). As the molecular weight falls, viscosity potential falls rapidly but the potential fermentability of the fibre increases (Bach Knudsen, 2016). Critically, when molecular weight is reduced to the point that the fibre is now an oligosaccharide with degree of polymerization 6 or less, depending on their structure, these molecules can be absorbed directly by many species and some act to markedly upregulate production of oligosaccharide transporters and fibre degrading enzymes (Amir, 2021; Leth et al., 2023), an activity recently described as being stimbiotic (Gonzalez-Ortiz et al., 2019).

### 2.3 Backbone structure

The identity and the linkage between the monosaccharides defines not only the name of the fibre but also its characteristics – viscosity and fermentability for example (Bach Knudsen, 2014). Fructans and β-1,3-1,4-glucans for example are more easily fermented than β-1,4-arabinoxylans and cellulose, and the glucans and arabinoxylans have the potential to be far more viscous than the fructans and cellulose (Bach Knudsen, 2014).

### 2.4 Degree of branching

Polysaccharides have to be depolymerised so that they become small enough (e.g. oligosaccharides or even monomers) to be absorbed and fermented by the intestinal microbiota. The success and speed of depolymerisation initially depends upon endo acting enzymes randomly cleaving the interior of the polysaccharide at sites which conform to its binding requirements. The catalytic ability of these enzymes is reduced if there is excessive “cloaking” of the backbone by substitutions of sugars, uronic acids or phenolic compounds. As a result, the greater the degree of branching and the more complex the branching structures projecting from the backbone, the slower the progress made and the less fermentable the fibre (Vangsøe et al., 2019, 2021).

### 2.5 Insoluble particle size

Although this is discussed later, it is prudent to mention here that the majority of fibre in the diet is in the form of insoluble, particulate material, which ranges considerably in size. Typical feeds contain particles ranging from 2 mm down to 100 μm which fundamentally differ in their interactions with the gizzard, small intestine (passage rate) and caeca (whether they enter or not). Such particles are not inert and can absorb considerable amounts of water, but they are not rapidly fermented (Choct, 2015a, Choct, 2015b). They do, however, provide a reservoir for the release of additional soluble material as they transit through the gut and get exposed to exogenous or microbiota derived non-starch polysaccharides-degrading enzymes.

### 2.6 Section summary

The principal conclusion from the above is that fibre is a term used to describe an incredibly complex and heterogenous group of structures which differ markedly in the effects they have on the environment of the intestinal tract. Even small variations in composition can have significant biological effects. As a result, the description of a fibre source needs to be in much greater detail if we are to fully understand the benefits and the limitations that any one source can bring to the diet. At present, nutritionists tend to calculate the fibre contents of the diets they formulate but they rarely change formulations on the basis of the numbers they generate.

More advanced analytical methods which routinely enable nutritionists to understand the composition, size, degree or branching and solubility of the fibre which directly relate to its functionality are needed to allow its full potential to be realised (Schäfer et al., 2019).

## 3 The role of dietary fibre in the functional development of the gut

### 3.1 Functional effects of fibre on gut development

Fibre may affect gut development either through its physical structure or indirectly through its fermentability. The former is related to direct effects of the physical structure, for example large particulates can stimulate gizzard development, while the latter in addition is related to the interaction of fibre with the microbiome. Fibre may have many more delicate effects, but only the most conspicuous effects related to gizzard and caeca development will be covered here.

### 3.2 Effect of fibre on gizzard development

The proventriculus and gizzard are the true stomach compartments of birds, where hydrochloric acid and pepsinogen are secreted by the proventriculus and mixed with the ingesta by muscular movements in the gizzard. The gizzard also plays an important role in grinding feed material. Thus, the gizzard consists of strongly myelinated muscles and has a koilin layer which due to its sand-paper like surface will aid in the grinding process. Grinding activity and the regulation of this activity in the gizzard has been described in detail by Duke (1992).

It has been shown repeatedly that when structural components such as whole or coarsely ground cereals or coarse fibre materials such as hulls or wood shavings are added, the gizzard increases in size and the pH of the gizzard content decreases by a magnitude of 0.2 to 1.2 units (Huang et al., 2006; Jimenez-Moreno et al., 2009; Sacranie et al., 2012; Svihus et al., 2013a, Svihus et al., 2013b). The logical explanation for the increased size of the gizzard is stimulation of muscular activity to grind down coarse particles, to allow them to pass through the pyloric sphincter to the small intestine. This leads to ingesta being exposed to greater quantities of hydrochloric acid and for a longer time period. Since feed usually has a pH close to neutral, high feed intake can be expected to result in an elevated gizzard pH unless gastric juice secretion is able to increase in accordance with intake. This is probably the main reason why gizzard pH is reported to be higher with pelleted diets when compared to mash diets (Engberg et al., 2002; Huang et al., 2006; Frikha et al., 2009), although less structure due to the grinding effect of pelleting will also contribute to this effect (Engberg et al., 2002; Svihus et al., 2004). As reviewed extensively by Svihus (2011), the increase in size of the gizzard when the diet contains structural components in the form of coarse fibre or cereals improves digestive function both through an increased retention time, a lower pH and a better grinding. However, a better regulation of feed flow may also be an important cause for such an effect.

As observed before in experiments with turkeys (Jackson and Duke, 1995), refeeding of birds which have been starved to empty will result in the entire length of the small intestine becoming filled with contents within an hour. Experiments have demonstrated that birds with an underdeveloped gizzard due to lack of structural components in the form of the insoluble fibre oat hulls (compared with a finely ground cellulose control) will allow for a very rapid flow of feed material into the small intestine when starved and then refed (Sacranie et al., 2017; Itani and Svihus, 2019). Although the flow is surprisingly fast independent of gizzard function, the amount that bypassed the gizzard was observed to be considerably larger for birds without a well-functioning gizzard. Even more conspicuously, the starch content in the ileal material was much higher for these gizzard-compromised birds. In fact, the starch content in the ileal material 1 h after refeeding suggested it was virtually nondigested feed material, with a starch content of between 30% and 40% of the dry matter (Sacranie et al., 2017). Poor digestibility of the nutrients at the ileal level were largely compensated for at the faecal level, suggesting that gizzard-compromised birds lost significant amounts of dietary nutrients to their large intestinal microbiota. However, the analysis of the ileal contents was performed only 1 h after feeding, and it is unlikely that all of the nutrients would have passed to the caeca or colon in such a short period of time. Hence, once an equilibrium is established, the differences between the gizzard-compromised and oat-hull fed birds would likely diminish at the ileal level. Since Kadhim et al. (2011) have shown that the ileum has a considerable amylase activity and Ferrer et al. (1994) has demonstrated a considerable absorption capacity of the ileum, one possibility is that digestion and absorption continue through the whole small intestine. Another possibility is that reflux allows for digestion and absorption in more anterior parts of the small intestine. Fibre can physically interact with the intestine and stimulate more violent peristalsis and anti-peristalsis which would enhance this process. The role of the gizzard as a feed flow regulator may indicate that stimulation of the gizzard by coarse fibre would affect feed intake, i.e. that coarse fibre would reduce feed intake.

However, no such effects have been observed in experiments with isonutritious diets where finely ground and coarsely ground fibre has been compared (Sacranie et al., 2012; Svihus et al., 2013a, Svihus et al., 2013b; Itani and Svihus, 2019). In fact, Sacranie et al. (2012) even observed an increased feed intake when coarse oat hulls were used instead of ground oat hulls. It may be that a greater retention time in the gizzard results in the exiting digesta requiring far less digestive effort in the small intestine, allowing for more rapid passage through the rest of the tract. As long as the gizzard is large enough to accommodate this increased gastric retention, the total feed flow, i.e. intake, can be maintained and may even increase.

One important factor is the form of the feed, which to a large extent will determine feed intake. Pelleting of the diet will usually increase feed intake of broiler chickens by 10% to 20% (Engberg et al., 2002; Svihus et al., 2004), and thus will increase the demands on an already high-performing digestive system. An increase in digestibility when diets were given as mash compared to as pellets was observed by Svihus and Hetland (2001) and indicates that pelleting may cause an overload of the digestive system. Engberg et al. (2002) found significantly higher levels of digestive enzymes when diets were given as mash compared to pellets, and also that pelleted diets resulted in a much more poorly developed gizzard than when mash diets were given. Thus, since the gizzard probably has an important role as a feed-flow regulator, it is possible that the combined effect of a high feed intake and a low gizzard-stimulating effect due to lack of coarse fibre increases the risk of passage of material through the digestive tract faster than it can process it. This fits with conclusions made by Rougière and Carré (2010), who based on passage studies concluded that retention time in the proventriculus/gizzard was a major limiting factor for digestion in broiler chickens. Increased feed intake due to pelleting may therefore have particularly detrimental effects when there are no structural components in the diet and therefore a small and underdeveloped gizzard. Perhaps structural components in the diet are therefore even more important for pelleted compared with mash diets. Environmental conditions may be important in this context, since birds will to some extent compensate for lack of structural components in the diet by eating litter materials such as wood shavings if available (Hetland et al., 2005; Hetland and Svihus, 2007).

### 3.3 Effect of fibre on caeca development

In studies of wild avian species, it has been shown that the caeca are highly adaptive, and will change in size according to the nature of the diet. In willow ptarmigan, the length and weight of the caeca have been shown to increase dramatically as the birds adapt to a fibre-rich winter diet (Fenna and Boag, 1974; Pulliainen and Tunkkari, 1983). Pulliainen and Tunkkari (1983) thus found the caeca to be more than 30% longer in the winter than in the summer. The time needed for caecal adaptations to take place is uncertain. According to Redig (1989), 2 to 3 months are required for the caeca to adapt to a new diet. Duke et al. (1984) observed that the caeca were 25% longer and contained twice the amount of dry matter for turkeys after adaptation to a high-fibre diet (40% oat hulls, 15% wheat middlings and 10% alfalfa) for 4.5 months. However, this does not fit with observations in broiler chickens, where significant changes in size of the caeca and fermentative activity have been observed after only a few weeks on a diet providing more fermentable (e.g. soluble glucans, low complexity fibre components and pentose sugars) material to the caeca (Longstaff et al., 1988; Jorgensen et al., 1996; Jozefiak et al., 2006; Rehman et al., 2007). Jozefiak et al. (2006), for example, observed a significant increase in both the weight of the empty caeca and the contents of the caeca when a barley diet, providing a larger quantity of soluble fibre, was offered for 5 weeks instead of an oat-based diet which was much lower in soluble but higher in insoluble fibre. Beta-glucanase addition did not affect caeca weight. Similarly, Rehman et al. (2007) observed a significant increase in both weight of the caeca and weight of the contents after 5 weeks with 1% inulin added to the diet. Longstaff et al. (1988) even found significant changes in weight and length of the caeca in broiler chickens only two weeks after inclusion of pentose sugars or uronic acids.

From the above, it is clear that fibre will potentially affect caeca development through being passed into the caeca. However, only solubilized fibre particles, either found in the diet or as a product of exogenous enzymes or microbial fermentation, will be able to pass through the narrow opening between the colon and the caeca, which is further constricted by villi projecting into the mouth of the junction. Although the effects of stimulating caeca development remains unclear, a certain production of energy-yielding short-chain fatty acids (SCFA) as well as an improved water-reabsorption has been indicated as potential beneficial effects (Svihus et al., 2013a, Svihus et al., 2013b).

## 4 Antinutritional effects of NSP

Non-starch polysaccharides have a range of antinutritional effects in both pigs and poultry but the relative influence of these varies between them (Moran and Bedford, 2022). The major sources of the antinutritional effects of NSP include altered chyme viscosity in the lumen, mechanical abrasion of the mucosa, bulk density, water holding capacity and feed intake constraints. The so-called “cage effect” that limits access of endogenous enzymes to cell contents and inconsistent effects on the enteric microbiome (Bedford and Cowieson, 2012; Choct, 2015a, Choct, 2015b).

That high molecular weight soluble NSP can increase the viscosity of lumen contents has been known for several decades (Choct and Annison, 1990). Bedford and Classen (1992) reported a significant increase in chyme viscosity in broiler chickens fed graded concentrations of rye and noted a decrease in viscosity when a pentosanase was applied. It is now well accepted that soluble, high molecular weight NSP can induce changes in the viscosity of the gut of poultry (and to a lesser extent swine, given important anatomical differences, the lower dry matter content of swine digesta and variance in particle size distribution of digesta). These changes result in a series of complications involving diffusion of nutrients, rate of passage of digesta and changes in the fermentation patterns in the microbiome. For example, diets with a high concentration of highly viscous polysaccharides may result in microbiological changes that lead to deconjugation of bile salts and interfere with fat emulsification and digestion (Bedford and Cowieson, 2012). These microbiological effects will be explored in more detail in a later section.

In addition to inducing changes to the viscosity of lumen contents, NSP can directly influence the flow of endogenous nutrients from the ileum of broilers. Angkanaporn et al. (1994) noted a significant increase in the flow of endogenous amino acids from the ileum of broilers when wheat arabinoxylans were fed in a bolus. Interestingly, similar negative consequences were not noted when either cellulose or a polyethylene polymer (alkathene) was fed. Specifically, total endogenous amino acid flow in the ileum of broilers was increased from 30.2 g/kg dry matter intake in the control group to 40 to 54 g/kg dry matter intake in the groups receiving wheat NSP. The lack of response to cellulose, which was equally if not more insoluble and particulate than the wheat NSP, suggests that mechanical abrasion of the mucosa was not the primary mechanism and instead these responses may be induced by an increase in viscosity and a reduction in feed passage rate, very likely involving the hind gut microbiome (Bedford and Cowieson, 2012; Choct et al., 2006).

Instructively, Cowieson et al. (2015) noted that the concentration of high molecular weight NSP in wheat may have been reduced over the past two decades, likely due to plant breeding intervention which likely was not deliberately targeting NSP content. In this work, the authors noted that soluble and insoluble arabinose + xylose in wheat in 2015 was around 9 and 50 g/kg dry matter which contrasts with values of 16 to 18 and 60 to 63 g/kg dry matter, respectively, reported in 1997 and 2006 (Bach Knudsen, 1997; Choct, 2006). These trends suggest that viscosity per se may be of lesser influence today than it was in the 1990s and 2000s and this in turn suggests that alternative mechanisms such as those more directly involving xylo-oligosaccharides and the microbiome, are worth exploring in more detail. Indeed, a certain critical concentration of soluble NSP may be beneficial rather than act as an anti-nutrient though the thresholds for tolerance and the need to prime the microbiome to generate adequate complements of extra-cellular enzymes is not clear (Bedford and Apajalahti, 2022). Nguyen et al. (2021b) noted that although ileal *Lactobacillus* numbers in broilers increased with each increment in dietary soluble NSP, viscosity increased and performance was compromised (Nguyen et al., 2022) suggesting a threshold does exist, particularly for younger birds. It is likely that there is a balance between optimizing feed intake, feed passage rate, minimising endogenous nutrient loss and creating an optimal taxonomic and functional profile in the hind-gut microbiome. Cowieson et al. (2015) also observed a reduction in the intestinal flow of fucose (a biomarker for mucin) in response to added xylanase and a negative correlation between the ileal flow of fucose and the digestibility of both nitrogen and energy. This is further evidence of a role of NSP and exogenous xylanase on endogenous nutrient flow which should be considered in parallel to beneficial effects mediated via the microbiome.

Helpfully, there have been several recent developments in rapid analytical assays NSP speciation and quantification. Aureli et al. (2017) published results showing successful near-infrared reflectance spectroscopy calibrations had been developed for a wide range of nutrients including total and phytate phosphorus and these were updated more recently with additional data from Nieto-Ortega et al. (2022) on a variety of soluble and insoluble NSP constituent sugars. Databases and rapid assays of this kind help nutritionists navigate the fibre space and will be a critical part of the toolbox required to establish an equilibrium between avoiding unintended antinutritional effects and leveraging the role of the enteric microbiome more fully. This latter domain will be further elucidated in the following section.

## 5 Interaction between dietary fibre and the enteric microbiome

### 5.1 Fibre as a substrate

The structure of fibre may be broken down to some degree by the host but is not absorbed. Large proportions can, however, be fermented (as much as 40%) and the products of fermentation utilised by the host. The structure and composition of the fibre determines both the numbers and species distribution of the inhabitants of the caecum and to some extent the small intestine. As noted above, the fermentability of fibre decreases as its size and complexity increases, with some backbone structures being more rapidly fermented than others. In effect, the small intestinal populations are more adapted to utilising the rapidly fermented, small and much simpler fibre structures which includes simple sugars and some oligosaccharides (Apajalahti and Rinttila, 2019; Bedford and Apajalahti, 2001). Some recent data has suggested that even small arabino-xylooligosaccharides can be fermented in the terminal ileum (Bautil et al., 2020). As a result, the supply of such simple sugars and oligosaccharides can influence the populations and end product of fermentation in the ileum. In the caecum, a great deal of work has shown that supply of various fibre types can significantly influence population structures and density which ultimately influences SCFA amounts and proportions (Courtin et al., 2008; Eeckhaut et al., 2008; Jozefiak et al., 2006). This in turn can have profound effects on gut and host health. Entry into the caeca is generally limited to soluble fibre and very small particulates and since the caeca is where the majority of fermentation takes place, consideration of these restrictions is essential if caecal fermentation is to be optimised. As the bird ages and the fermentative capacity of the small intestine increases, the supply of fermentable nutrients to the caecum is restricted to the progressively more intransigent fibre sources and the population adapts accordingly (Moran and Bedford, 2022). With age the caeca also become more active and fibre fermentation increases (Bautil et al., 2019) such that adequate supply of fermentable fibre may be a consideration, particularly in corn soy-based diets which are limited in their soluble fibre content. Indeed, addition of more fermentable fibre sources to corn-soy or sorghum-soy based diets has been shown to augment caecal fermentation and some have suggested formulating to a minimum soluble NSP content (Nguyen et al., 2022; Singh et al., 2021) to optimise intestinal health. Limitations on universal availability of rapid measures of soluble fibre content of the diet will hinder implementation of this concept, although some near infrared calibrations are becoming available as noted earlier.

### 5.2 Fibre as an antinutrient

High molecular weight arabinoxylans and β-glucans which are particularly present in rye, triticale, wheat, barley and oats can induce significant increases in intestinal viscosity which can impede the digestive process digestion. As noted earlier, modern day cultivars of wheat and barley contain far less viscous polysaccharides than those available in the 1990s when much of this work was undertaken and as a result some of the extremes noted back then likely will not be encountered today. Nevertheless, even moderate increases in intestinal viscosity will reduce nutrient digestibility, and in particular that of fat and fat soluble nutrients due to reduced emulsification capacity in viscous digesta (Pasquier et al., 1996) and the fact that viscous digesta encourages increased microbial growth in the small intestines and particularly of species capable of degrading bile salts (Hubener et al., 2002; Maisonnier et al., 2003; Smits et al., 1998). Reduced nutrient removal by the host consequently delivers more substrate to the large intestine where it can support excessive growth by potential pathogens. An extreme example of such an effect was noted in 1996 when a viscous wheat-derived arabinoxylan was added to a sorghum, soybean and meat and bone meal-based diet at 6.6% (Choct et al., 1996). The extract markedly increased ileal viscosity which reduced digestibility of all nutrients and increased volatile fatty acid (VFA) production presumably as a result of delivering significant amounts of readily fermentable starch and protein to commensal bacteria in the ileum. Interestingly, when an enzyme was applied which reduced viscosity dramatically, not only did ileal fermentation return to that of the control, but caecal VFA levels increased by almost 300% (Choct et al., 1996). The authors suggested that in its native state, the extract was so viscous that it aggregated into suspensions that were too large to enter the caeca. On depolymerisation by inclusion of the enzyme a considerable amount of arabinoxylan was immediately made available for fermentation, which suggests that even in wheat-based diets caecal fermentation may be limited by substrate entry and that some soluble fibre may aggregate and become be too large to enter the caeca.

### 5.3 Fibre as a stimulant

Recent work has suggested that the use of enzyme to produce oligosaccharides from complex fibre, or the addition of such oligosaccharides directly to the diet can markedly alter that activity of the resident caecal and perhaps even ileal microbiota (Bautil et al., 2020; Bedford and Apajalahti, 2018; Cardoso et al., 2020; Ribeiro et al., 2018; van de Wiele et al., 2008). This effect has been noted where the concentration of oligosaccharide used is so low, for example 100 g/tonne (Ribeiro et al., 2018), that it could not possibly contribute meaningfully to SCFA production directly and thus the effect noted must be due to a change in microbiota metabolism. Indeed, feeding xylo-oligosaccharides has been shown to accelerate total arabinoxylan “digestibility” (Bautil et al., 2020; Ribeiro et al., 2018) and considerably enrich the caecal microbiota proteome relating to fibre degrading enzyme and fibre-oligosaccharide transporters (Amir, 2021). The term “stimbiotic” has been suggested (Gonzalez-Ortiz et al., 2019) to describe this effect as a response to “dietary additives that are able to stimulate a fibre-degrading microbiome to increase fibre fermentability at doses which clearly are too low to contribute in a meaningful manner to VFA production directly”. Recent work has suggested that many bacterial species rely on recognising the presence of specific oligosaccharides which they can internalise and metabolise to gain a competitive advantage (Leth et al., 2023).

### 5.4 Starch as a fibre

In some cases, resistant starch, which in itself is included in the dietary fibre estimation in some cases, can form an appreciable amount of caecal fermentable material. The amount of starch which reaches the caeca is dependent upon the rate of its digestion in the small intestine (Kim et al., 2020) which can be related to cereal sample, starch structure and degree of protein encrustation, viscosity (as noted above) and drying and processing conditions (Moran, 2019). If significant quantities of resistant starch reach the caeca, then its rapid fermentation to lactic acid can create problems for the host since this not only impedes fermentation of other fibre sources but may also create an acidotic like condition.

### 5.5 Nutritional benefits of exogenous carbohydrases

The nutritional benefits of exogenous carbohydrases vary depending on individual product recommendations, animal species and growth stage, diet type, enzyme dose and the presence of additional feed enzymes such as phytase or protease (Cowieson and Bedford, 2009; Cowieson et al., 2010; Cowieson, 2010). However, typical nutrient release values for NSP degrading enzymes would include a metabolizable, digestible, or net energy value of 50 to150 kcal/kg and a modest digestible amino acid value of 1% to 3%. The energy release values are usually higher in diets based on wheat, barley, oats, rye, and triticale and lower in diets based on corn or sorghum. This is principally associated with the lower concentration of high molecular weight soluble pentosans in corn and sorghum (Bach Knudsen, 1997) and axiomatically, the relatively lower metabolizable energy concentration of wheat compared with corn though particle size and animal age play important roles (Amerah et al., 2008; Khalil et al., 2021).

One of the most important factors that can promote or demote the mean nutrient release value of carbohydrase (and indeed other feed enzymes) is the relative nutrient digestibility in the control group (Cowieson and Bedford, 2009; Cowieson, 2010; Cowieson and Roos, 2014). In a meta-analysis of the effect of carbohydrases on the ileal digestibility of amino acids in pigs and poultry, the authors noted that around 65% of the variance in enzyme effect size could be explained by the digestibility of amino acids in the control diet (Cowieson and Bedford, 2009). Indeed, the effect of carbohydrase on amino acid digestibility declined by around 50% for every 10% increase in the digestibility of the control group. Furthermore, this relationship has also been confirmed for exogenous protease (Cowieson and Roos, 2014) and exogenous phytase (Cowieson et al., 2017a, Cowieson et al., 2017b). Importantly, the association between the relative digestibility of the control diet and the magnitude and consistency of nutrient release by feed enzymes is not linear but typically follows a second order polynomial curve (Cowieson and Bedford, 2009). This implies that an increase in the digestibility of the control group from e.g. 70% to 80% will create a relatively more substantial headwind for enzyme effect size than a change from 80% to 90%. A similar relationship between the inherent digestibility in the control diet and enzyme effect size has been noted for energy metabolism. For example, Choct et al. (2004) observed an increase in the apparent metabolizable energy of a “normal metabolizable energy wheat” from 13.7 MJ/kg to around 14.2 MJ/kg (an increase of 0.5 MJ/kg or around 3.6%) but in a “low metabolizable energy wheat” from 12.7 to 13.7 MJ/kg (an increase of 1 MJ/kg or around 7.8%). Douglas et al. (2000) noted an equivalent relationship between the inherent ileal digestible energy of soybean meal and the effect of a blend of xylanase, glucanase and protease on the same. Soybean meal with a low digestible energy responded more readily to the supplemental enzyme that soybean meal with a high digestible energy value, with an enzyme response range from −174 to +299 kcal/kg and an average of +56 kcal/kg. Thus, the use of generic or static matrix values for carbohydrases for either energy or amino acids will generate variable outcomes in carcass composition and animal live performance phenotypes. Most precise estimates may be delivered by articulating raw material quality surveillance with feed enzyme selection and dosing (Cowieson, 2010), for example, via the use of near-infrared spectroscopy or similar laboratory testing.

Finally, in addition to variation in the inherent nutritional quality of the control diet and relative substrate concentrations, the animals that receive the diets are also influential in the digestibility values that are returned. Animals with a lower digestive capacity, more sensitivity to dietary antinutrients, poor disease or general health status or with compromised immune or microbiome function or exposed to environmental or other stressors will typically deliver below average digestibility values and may, in turn, benefit more from exogenous feed enzymes. For example, Cowieson et al. (2020) noted that a single soybean meal fed to 18 individually housed broilers returned a standardised amino acid digestibility ranging from 54% to 80%. Similar between-bird variance in energy metabolism and starch digestibility has been reported in the past for broilers fed wheat (Choct et al., 1999) and this was confirmed recently in maize-based diets (Bassi et al., 2023).

It can be concluded that exogenous carbohydrases reliably generate increases in the metabolizable or digestible energy value of diets and also have the capacity to increase amino acid digestibility and modify animal live performance. However, substantial heterogeneity exists in these responses, and this is associated largely with the inherent nutritional value of the diet or feed ingredient to which the enzymes are added. Systematic analysis and reporting of raw material quality, substrate concentrations and awareness of the relative health and nutritional status of the cohorts of animals that will receive the diets will bring more precision in enzyme selection, dosing and nutrient release value assignment.

## 6 Health benefits of exogenous carbohydrases

### 6.1 Reduction of antinutritive effects

The previous sections identify the potential for some components of dietary fibre to be anti-nutritive. In this regard exogenous carbohydrases perform multiple roles which ultimately have health giving benefits for the host. The activity of these enzymes likely loosens fibre structures and facilitates reduction of particle size in the gizzard provided the diet has been stored in the crop prior to entry into the proventriculus gizzard. This will facilitate more rapid digestion of cellular contents and as a result deprive the microbiota of nutrients which the host can utilise directly. Depolymerisation of viscous fibre structures also accelerates nutrient removal from the intestine by the host and again reduces the potential for excessive microbial growth in the small intestine. It also results in improved miscibility of the contents of the intestinal trace which increases the oxygen content of the digesta, providing a further barrier to invasion by opportunistic facultative anaerobic pathogens (Moran and Bedford, 2022). In this regard, exogenous carbohydrases reduce the challenge posed by that portion of fibre which is detrimental to animal health.

### 6.2 Provision of beneficial effects

Exogenous carbohydrases can produce smaller fibre fragments from the larger, more anti-nutritive fractions, and may even produce stimbiotic oligosaccharides directly. In essence they result in a progressive movement of fibre from the insoluble to the smaller soluble fractions. This can be very significant, particularly when antibiotics are not routinely administered since many intestinal resident bacteria preferentially ferment carbohydrate and produce SCFA which are beneficial for intestinal health and energy status, or, if fibre is limiting, putrefy protein to amines and indoles which are detrimental to intestinal and ultimately host health (Apajalahti and Vienola, 2016). This becomes more of an issue as the bird ages and the small intestinal microbiota matures, removing the most rapidly fermentable fibre prior to it reaching the caeca, thereby restricting caecal carbohydrate supply. Use of carbohydrases increases the supply of fermentable NSP, ultimately through dissolution and depolymerisation of insoluble NSP, thereby reducing the likelihood of protein putrefaction taking place.

Some exogenous carbohydrases are able to reduce soluble fibre to oligosaccharides which may have direct stimulatory activity on the fibre degrading microbiota. As noted in section 5, such stimbiotics (Gonzalez-Ortiz et al., 2019) can radically alter the ability of the resident microbiota to attack and ferment the fibre resident in the caeca, ultimately increasing the amount of SCFA produced and likely improving intestinal integrity and health.

## 7 Future opportunities in management of coarse insoluble fibre for poultry production

The positive effect of coarse insoluble fibre exerted through stimulation of gizzard functionality indicates that a certain level should be added to poultry diets. However, the level of fibre needed is still unclear, and should be targeted in future research. Adding to the complexity of this issue is that both the chemistry of the insoluble fibre and the content of other components such as coarse cereal particles affect the gizzard stimulating effect. Jimenez-Moreno et al. (2013), for example, observed a much stronger gizzard-stimulating effect when the insoluble fibre was in the form of oat hulls than when it was in the form of soybean hulls. This may be due to differences in structural rigidity and resilience to grinding, although this is far from certain. Further, as the level of oat hulls in the diet increased above 5% of the diet, no further increase in relative gizzard weight occurred, indicating that 5% was enough when this fibre source was used. Another example of the complex interaction between different sources of insoluble fibres was shown by Jimenez-Moreno et al. (2019), where both oat and sunflower hulls resulted in a dose dependent increase in gizzard size when both were included at 2.5% and 5% but oat hulls were much more effective at stimulating gizzard development than sunflower hulls. However, rice hulls had a similar gizzard-stimulating effect as oat hulls at 2.5% inclusion, but no further increment in gizzard size was noted with further increments in rice hull inclusion, in contrast to the effect noted for both oat and sunflower hulls. Similarly, the effect of insoluble fibre will very much depend upon the coarseness of cereal particles in the diet. Although the relatively fine grinding and the additional grinding effect of the pelleting effect would probably result in an insufficient gizzard stimulation by coarse cereal particles in most diets, more drastic feeding practices such as the use of whole wheat would certainly diminish the beneficial effect of insoluble fibre. Thus, there seems to be an unexplored possibility for inclusion of a certain level of insoluble fibre in poultry diets. However, further studies are needed to set specific targets for different fibre sources, where the coarseness of the cereal used in the diet is also taken into consideration. Taylor et al. (2021) demonstrated that broiler chickens given pelleted diets were able to maintain weight gain through an increased feed intake when oat hulls levels increased to as high as 30% of the diet. Thus, when sources of insoluble fibres are available, the risk of oversupplying the diet with these fibres at least seems to be low.

## 8 Conclusions

Fibre is the macro-ingredient in poultry diets for which there are the most significant gaps in our understanding. The role of fibre in animal health, welfare, microbiome function, behaviour, nutritional status, gut physiology, and environmental sustainability are not entirely clear. However, the concept that fibre is a relatively simple nutritional diluent or even a feed component that influences feed intake or digestive dynamics in the gut of poultry, have been replaced with a much more nuanced understanding of the functional effect of fibre. Fibre has also been much more successfully fractionated analytically and rapid methods now exist that give nutritionists the information they need to begin formulating diets with constraints on strategically selected sub-sets of oligosaccharides. However, more research is needed to explicitly associate high value phenotypes with specific fibre composition and concentration and to create more consistent and reproducible effects of exogenous carbohydrases. Given the recent increases in attention on sustainability, it is likely that the concentration and complexity of fibre in the diets of poultry will increase over time as nutritionists turn to locally sourced raw materials and make more complete use of by-products and other novel feed ingredients. This, coupled with new insights related to the value of fibre on gut function, microbiome modulation, animal health and welfare, will create significant impetus to optimize the fibre fraction of feed. It is likely that exogenous carbohydrases will make a critical contribution to success in this next chapter of macro-nutrient optimisation of poultry diets.

## Author contributions

**M.R. Bedford:** Conceptualization, Writing – original draft, Writing – review & editing. **B. Svihus:** Conceptualization, Writing – original draft, Writing - review & editing. **A.J. Cowieson:** Conceptualization, Writing – original draft, Writing – review & editing.

## Declaration of competing interest

We declare that we have no financial and personal relationships with other people or organizations that can inappropriately influence our work, and there is no professional or other personal interest of any nature or kind in any product, service and/or company that could be construed as influencing the content of this paper.
